# Conserved vulnerability of SoxR underlies zinc-mediated redox disruption and synergistic killing in *Klebsiella pneumoniae*

**DOI:** 10.1128/mbio.01146-26

**Published:** 2026-07-29

**Authors:** Jie Feng, Zhenxiao Tong, Haopu Dai, Chengjian Wan, Yuhang Bao, Xiaolong Hua, Runyu Chen, Feng Liang, Wenyuan Ma, Yang Li, Jianghui Li, Kewei Li, Guoqiang Tan

**Affiliations:** 1 Laboratory of Molecular Medicine, Key Laboratory of Laboratory Medicine, Ministry of Education, School of Laboratory Medicine and Life Sciences, Wenzhou Medical University 214181, Wenzhou, Zhejiang, China; 2 Department of Clinical Laboratory, The First Affiliated Hospital of Wenzhou Medical University 89657, Wenzhou, Zhejiang, China; 3 Key Laboratory of Clinical Laboratory Diagnosis and Translational Research of Zhejiang Province, Wenzhou, Zhejiang, China; University of Texas at Dallas, Richardson, Texas, USA

**Keywords:** *Klebsiella pneumoniae*, SoxR, iron-sulfur cluster, zinc toxicity, oxidative stress, antibacterial synergy

## Abstract

The escalating threat of antimicrobial resistance in *Klebsiella pneumoniae* necessitates novel therapeutic vulnerabilities. Here, we identify the redox-sensing regulator SoxR as a specific target of zinc toxicity and reveal that SoxR regulatory networks differ fundamentally even among closely related Enterobacteriaceae. Integrated *in vitro* and *in vivo* analyses show that zinc competitively displaces the essential SoxR [2Fe–2S] cluster, causing protein destabilization and functional inactivation. Consequently, bacteria fail to activate the canonical soxS–sodA defense axis, rendering them hypersensitive to vitamin K3-induced superoxide stress. Building on this mechanism, we established a synergistic strategy combining zinc with vitamin K3. This combination elicited potent bactericidal activity against *K. pneumoniae* (including ciprofloxacin-resistant strains) and *Salmonella* Typhimurium, with a remarkably low propensity for resistance development. Furthermore, treatment significantly improved survival in a *Galleria mellonella* acute infection model without detectable host toxicity. Collectively, highlighting SoxR [2Fe–2S] cluster integrity as a critical vulnerability, this work extends metal–redox crosstalk-based antibacterial intervention from *Escherichia coli* to a clinically important pathogen and provides a conceptual framework for developing redox-targeting therapies against drug-resistant gram-negative bacteria.

**IMPORTANCE:**

*Klebsiella pneumoniae* is a major drug-resistant pathogen whose survival in the host depends heavily on its ability to withstand oxidative stress. While the SoxR/SoxS system is well characterized in *Escherichia coli*, whether SoxR functions similarly in *K. pneumoniae* and whether it can be selectively targeted have remained unknown. Here, we show that the SoxR regulatory network displays clear species-dependent features: sequence comparison reveals low homology of *soxS* between the two organisms, and SoxR function cannot be simply interchanged across them. Importantly, we demonstrate for the first time that Zn²^+^ specifically targets *K. pneumoniae* SoxR by directly and competitively disrupting its essential [2Fe–2S] cluster, leading to protein instability, loss of redox regulatory function, and heightened sensitivity to oxidative stress. This previously unrecognized metal–redox interference mechanism exposes a conserved biochemical vulnerability in oxidative defense and provides a mechanistic basis for developing nontraditional antibacterial strategies that exploit intrinsic stress-response weaknesses rather than conventional growth targets.

## INTRODUCTION

*Klebsiella pneumoniae* is a representative gram-negative opportunistic pathogen that causes a wide spectrum of clinical diseases, including pneumonia, sepsis, urinary tract infections, and intestinal colonization, and has become one of the leading causative agents of hospital-acquired infections worldwide (1, 2). In recent years, the continuous emergence of multidrug-resistant and hypervirulent strains has been accompanied by a sustained increase in infection-associated morbidity and mortality caused by *K. pneumoniae*, thereby posing substantial challenges to clinical treatment (3–5). Against the backdrop of the escalating global antimicrobial resistance crisis, *K. pneumoniae* has been designated by the World Health Organization as one of the most threatening priority resistant pathogens, with carbapenem-resistant and third-generation cephalosporin-resistant Enterobacteriaceae classified as “critical priority” pathogens, highlighting the urgent need for novel intervention strategies (6). Large-scale epidemiological and genomic studies have further revealed that *K. pneumoniae* accelerates the co-evolution of antimicrobial resistance and virulence through the coordinated acquisition of plasmid-mediated resistance determinants and hypervirulence factors, facilitating its widespread global dissemination (7, 8). Collectively, these findings indicate that *K. pneumoniae* has emerged as a major public health threat characterized by high levels of antimicrobial resistance, virulence, and transmissibility, underscoring the limitations of conventional antibacterial strategies and the necessity to identify actionable targets from alternative biological perspectives (9).

Within the host, successful colonization and persistent infection by *K. pneumoniae* depend not only on its capacity to acquire and disseminate antimicrobial resistance but also critically on its ability to withstand innate immune pressures (10, 11). Among these pressures, oxidative stress mediated by reactive oxygen species (ROS) represents one of the central bactericidal mechanisms employed by host phagocytes against gram-negative pathogens (12), involving potent oxidants such as superoxide anions and hydrogen peroxide that directly damage bacterial membranes, proteins, and nucleic acids (13). Accumulating evidence demonstrates that during pulmonary infection, bacteremia, and systemic infection, *K. pneumoniae* is continuously exposed to intense oxidative stress imposed by the host immune system and that its tolerance to oxidative stress is closely linked to *in vivo* fitness and pathogenic potential (14). To adapt to this hostile environment, *K. pneumoniae* has evolved a relatively comprehensive antioxidant defense system, including superoxide dismutases, catalases, and multiple enzymatic pathways involved in respiratory metabolism and redox homeostasis maintenance, which have been shown to play crucial roles in counteracting exogenous ROS, inflammatory conditions, and elevated temperature, thereby directly influencing bacterial survival and virulence potential (15, 16). In parallel, epidemiological and functional studies suggest that antimicrobial-resistant and hypervirulent strains are frequently associated with enhanced antioxidant capacity, conferring a survival advantage within macrophages and in host environments (17). However, despite the progressive identification of antioxidant-associated effector molecules in *K. pneumoniae*, a systematic understanding of how this pathogen rapidly senses oxidative cues, transduces signals, and precisely coordinates downstream defense responses under oxidative stress remains lacking. Existing studies indicate that oxidative stress can drive the remodeling of specific transcriptional regulatory networks and intersect with efflux pump activity and antimicrobial susceptibility; however, the corresponding regulatory landscape in *K. pneumoniae* remains far from complete (18, 19). This knowledge gap limits a comprehensive understanding of oxidative stress adaptation in *K. pneumoniae* and, importantly, hampers further exploration of redox homeostasis as a target for anti-infective intervention.

Among gram-negative Enterobacteriaceae, SoxR is regarded as one of the most canonical oxidative stress sensors, whose activity depends on an internal [2Fe–2S] iron–sulfur cluster and which regulates downstream defense responses by sensing changes in the intracellular redox state (20). Current knowledge regarding the molecular structure of SoxR, its mode of signal activation, and the SoxR–SoxS transcriptional cascade has been established almost exclusively based on the *Escherichia coli* model system (21). In this paradigm, SoxR senses superoxide anions and redox-cycling compounds through changes in the oxidation state of its iron–sulfur cluster, thereby inducing transcriptional activation of *soxS* and initiating the expression of a broad set of antioxidative, defensive, and resistance-associated genes (22, 23). However, the regulatory properties of SoxR are not fully conserved across different members of the Enterobacteriaceae. Previous studies have suggested that, even among phylogenetically closely related bacteria, SoxR-mediated transcriptional output, downstream regulatory networks, and modes of coupling with global regulators can differ substantially (24). Whether SoxR proteins from different Enterobacteriaceae members retain functional compatibility within heterologous regulatory contexts remains unclear, raising the possibility that SoxR-mediated oxidative stress regulation may exhibit pronounced species-dependent characteristics rather than simple functional conservation. In *K. pneumoniae*, although homologs of *soxR* and *soxS* are retained at the genomic level, the gene sets regulated under oxidative stress, the impact on antimicrobial susceptibility, and the functional positioning of this system within the global antioxidant defense network remain insufficiently characterized (18). Notably, *K. pneumoniae* exhibits more complex and species-specific regulatory features in antimicrobial resistance development, efflux pump regulation, and metal homeostasis maintenance, raising the possibility that SoxR participates in controlling efflux system expression and is linked to multidrug-resistant phenotypes (25, 26).

Iron–sulfur (Fe–S) proteins, owing to their unique metal-coordination properties, are highly sensitive to fluctuations in the intracellular metal environment and are widely regarded as key molecular platforms for bacterial sensing of redox stress and environmental challenges (27, 28). In *K. pneumoniae*, maintenance of transition metal ion homeostasis is essential for bacterial survival and pathogenicity, with zinc, as an indispensable micronutrient, supporting the function of numerous metabolic enzymes and regulatory proteins (29). Accumulating evidence indicates that disruption of zinc homeostasis not only compromises oxidative stress tolerance in *K. pneumoniae* but also markedly affects virulence and antimicrobial tolerance phenotypes (30). In addition to zinc, copper ions play a dual role in host–pathogen interactions, as excessive copper accumulation can impair bacterial viability by inducing oxidative damage, whereas *K. pneumoniae* relies on finely tuned copper homeostasis systems to counteract this stress (31).

In our previous series of studies, the inhibitory effects of metal ion overload on bacterial iron–sulfur (Fe–S) cluster biogenesis have been systematically elucidated, with *E. coli* serving as a model organism to define the underlying molecular basis. We first demonstrated that excessive intracellular copper ions directly target the Fe–S cluster assembly proteins IscA and IscU under both aerobic and anaerobic conditions. By competitively occupying their metal-binding sites, copper blocks [4Fe–4S] cluster biogenesis, leading to the inactivation of multiple Fe–S proteins and revealing an oxygen-independent core mechanism of copper toxicity (32, 33). Subsequent studies showed that zinc ions likewise bind to IscU, IscA, and ferredoxin, selectively inhibiting *de novo* Fe–S cluster assembly rather than disrupting preassembled clusters, thereby indicating a direct causal link between zinc toxicity and Fe–S cluster homeostasis imbalance (34). Building on this foundation, we recently uncovered a previously unrecognized metal–redox crosstalk mechanism in which zinc overload selectively disrupts the [2Fe–2S] cluster of the master oxidative stress regulator SoxR in *E. coli*. This disruption leads to SoxR inactivation and subsequent dysregulation of the SoxS–ZnuACB/SOD defense axis, thereby directly converting metal homeostasis imbalance into heightened oxidative stress susceptibility (35). This finding extends Fe–S cluster damage targets from metabolic enzymes and assembly proteins to the level of redox sensing and transcriptional regulation and further suggests that SoxR-dependent oxidative defense systems may represent a critical vulnerability of bacteria under metal stress.

In the present study, *K. pneumoniae* was selected as the research focus to systematically investigate the effects of zinc ions on the SoxR-mediated oxidative stress defense pathway. We demonstrated that SoxR in *K. pneumoniae* is likewise highly sensitive to zinc, as zinc overload markedly compromises SoxR functional stability and downregulates transcription of its key downstream targets *soxS* and *sodA*. These findings indicate functional evolutionary conservation of the SoxR-dependent superoxide defense axis among Enterobacteriaceae members. Furthermore, through integrated *in vitro* and *in vivo* analyses, we directly demonstrated structural instability of SoxR under zinc stress and, for the first time, proposed and validated a molecular model in which zinc ions competitively target the SoxR [2Fe–2S] cluster, leading to SoxR inactivation. This mechanism extends metal interference–induced oxidative stress imbalance to a clinically important pathogen. The combined application of zinc ions and the classical superoxide generator vitamin K3 synergistically amplifies redox homeostasis disruption, resulting in efficient antibacterial activity. This strategy exhibited pronounced antibacterial efficacy in both *in vitro* bacterial models and a *Galleria mellonella* acute infection model. Taken together with our parallel combination therapy studies in *E. coli*, these findings collectively indicate that targeting Fe–S cluster integrity and SoxR-dependent oxidative stress defense pathways may constitute a broadly applicable nontraditional antibacterial paradigm, providing new theoretical foundations and strategic directions for combating infections caused by drug-resistant gram-negative pathogens.

## RESULTS

### Zinc ions specifically target the [2Fe–2S] cluster of *K. pneumoniae* SoxR and reduce its stability in *E. coli* cells

Before investigating the biochemical effect of zinc on *K. pneumoniae* SoxR, we first asked whether the SoxR regulatory logic of *K. pneumoniae* is functionally compatible with the canonical SoxR–SoxS oxidative response system characterized in *E. coli*. As shown in Fig. S1A, in wild-type *Escherichia coli* MC4100, paraquat (PQ) robustly activated the transcription of the SoxR downstream genes *soxS* and *sodA*. In contrast, this response was completely abolished in the *soxR^-^* strain (Fig. S1B), confirming the SoxR dependence of this oxidative transcriptional program. Complementation of the *soxR^-^* strain with native *E. coli soxR* fully restored PQ responsiveness (Fig. S1C). However, strikingly, complementation with *soxR* from *Klebsiella pneumoniae* failed to restore PQ-induced activation of *soxS* or *sodA* (Fig. S1D). Consistent with this functional incompatibility, sequence comparison revealed that the *soxS* genes of *E. coli* and *K. pneumoniae* share relatively low homology (data not shown), suggesting that the SoxR regulatory network exhibits pronounced species-dependent characteristics and cannot be simply interchanged between these two Enterobacteriaceae species. These observations indicate that the SoxR regulatory logic of *K. pneumoniae* is not readily “compatible” with the *E. coli* transcriptional background, highlighting species-specific features of SoxR-mediated redox sensing. We next examined whether, despite this regulatory divergence, the biochemical vulnerability of SoxR to zinc might be conserved.

**Fig 1:**
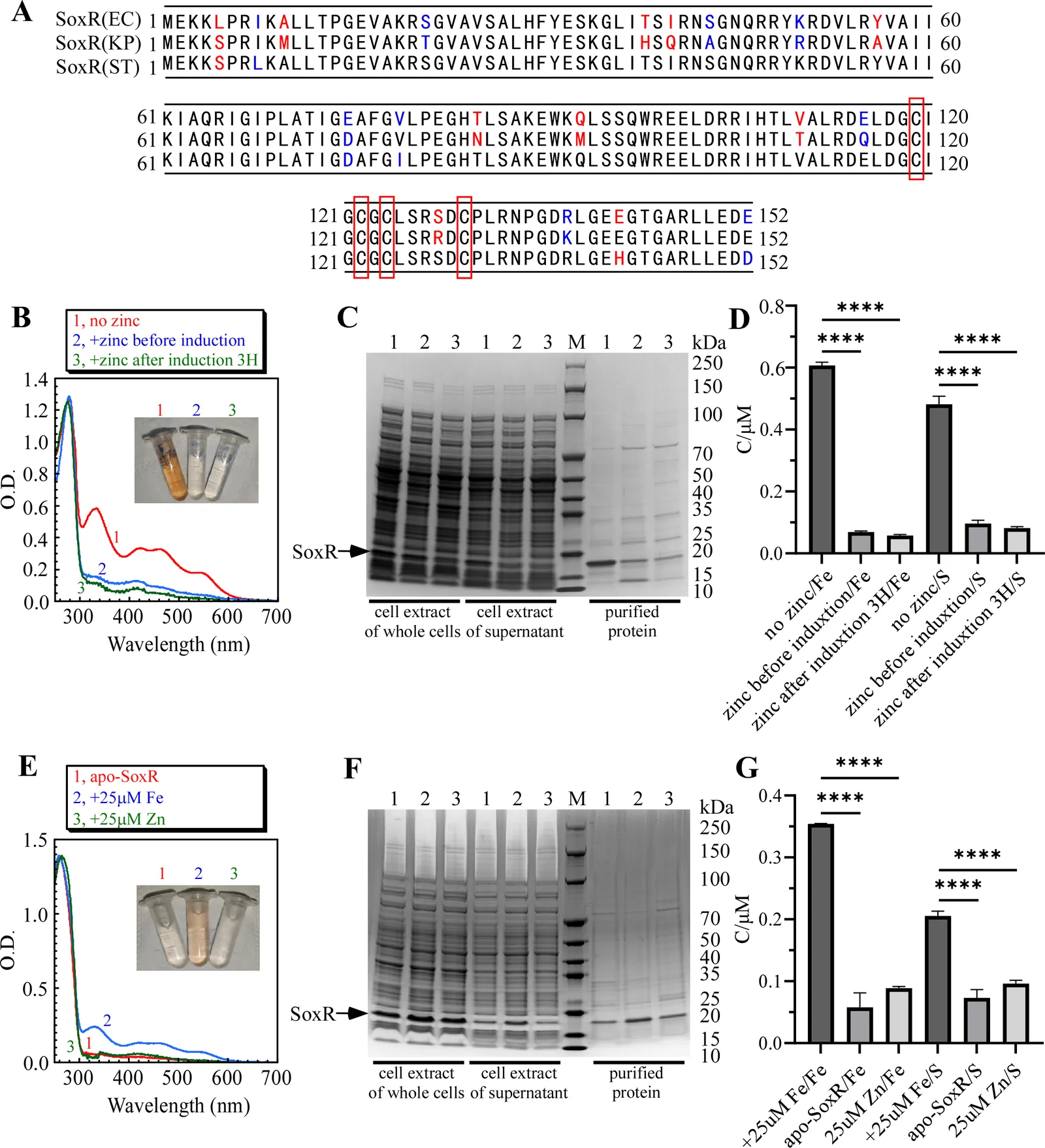
Zinc ions specifically target the [2Fe–2S] cluster of *K. pneumoniae* SoxR and reduce its stability in *E. coli* cells. (**A**) Amino acid sequence alignment of SoxR proteins from *E. coli*, *K. pneumoniae*, and *S*. Typhimurium. Residues shown in red indicate nonconserved amino acids, whereas residues shown in blue represent amino acids with similar physicochemical properties but not identical sequences. Red rectangles indicate the putative cysteine residues involved in [2Fe–2S] cluster coordination in all three SoxR proteins. (**B**) UV-visible absorption spectra of *K. pneumoniae* SoxR heterologously expressed and purified from *E. coli*. SoxR proteins purified under three conditions were compared: no zinc supplementation in LB medium (spectrum 1), addition of 1 mM ZnSO_4_ prior to induction (spectrum 2), and addition of 1 mM ZnSO_4_ after 3 h of induction (spectrum 3). Insets show representative images of purified SoxR proteins under the corresponding conditions. (**C**) SDS-PAGE analysis of *K. pneumoniae* SoxR expression and purification under different zinc treatment conditions. Whole-cell lysates, soluble fractions, and purified SoxR proteins obtained under each condition are shown. Arrows indicate the position of the SoxR protein band. (**D**) Quantitative analysis of iron (Fe) and sulfur (S) contents in purified SoxR proteins under different zinc treatment conditions. (**E**) UV-visible absorption spectra of SoxR proteins analyzed under simulated *in vivo*-like conditions using M9 minimal medium. Spectra were obtained from purified apo-SoxR (spectrum 1), SoxR reconstituted with 25 μM Fe (spectrum 2), and SoxR treated with 25 μM Zn (spectrum 3). Insets show representative images of the corresponding protein samples. (**F**) SDS-PAGE analysis of the intracellular stability of SoxR proteins subjected to different metal treatments in M9 minimal medium. Whole-cell lysates, soluble fractions, and purified SoxR proteins are shown. (**G**) Quantitative analysis of iron and sulfur contents in SoxR proteins under different metal treatment conditions in M9 minimal medium. All experiments were performed at least three times, and the most representative results are shown.

We first performed an amino acid sequence alignment of SoxR proteins from *E. coli*, *K. pneumoniae*, and *S*. Typhimurium. As shown in Fig. 1A, these SoxR proteins exhibited a high degree of overall sequence similarity, and the putative cysteine residues involved in [2Fe–2S] cluster coordination were highly conserved, suggesting that the mechanism by which zinc ions target SoxR may be conserved across species.

To test this hypothesis, we heterologously expressed and purified SoxR from *K. pneumoniae* in *E. coli* and compared its spectroscopic characteristics under different zinc treatment conditions. As shown in Fig. 1B, purified SoxR obtained in the absence of zinc exhibited characteristic UV-visible absorption peaks corresponding to a [2Fe–2S] cluster. In contrast, SoxR proteins purified either after supplementation with 1 mM ZnSO₄ prior to induction or after the addition of 1 mM ZnSO₄ following induction displayed markedly reduced Fe–S-specific absorption signals, indicating that zinc treatment substantially disrupted the Fe–S cluster status of SoxR. We subsequently analyzed SoxR expression and purification under different zinc treatment conditions by SDS-PAGE (Fig. 1C). The results showed that zinc treatment did not significantly alter the overall intracellular expression level of SoxR. However, compared with the control group, the amount of SoxR detected in the soluble fraction was markedly reduced in zinc-treated cells, and the overall purity of the purified protein was substantially decreased, suggesting that zinc ions reduced SoxR stability and promoted its precipitation. Further quantification of iron and sulfur contents revealed that zinc treatment led to a significant reduction in both elements within the SoxR protein (Fig. 1D), demonstrating that zinc ions specifically disrupted the [2Fe–2S] cofactor of *K. pneumoniae* SoxR.

To further assess whether this effect persisted under conditions more closely resembling the intracellular environment, we analyzed SoxR proteins in M9 minimal medium lacking iron and zinc ions. As shown in Fig. 1E, apo-SoxR purified without metal supplementation exhibited minimal Fe–S-specific absorption signals, whereas supplementation with exogenous iron enabled the formation of a predominant SoxR species displaying pronounced Fe–S cluster features. In contrast, SoxR proteins supplemented with zinc remained devoid of characteristic Fe–S absorption peaks. Consistently, SDS-PAGE analysis demonstrated that, in M9 medium, SoxR supplemented with iron existed in a relatively stable intracellular form, whereas SoxR proteins obtained under metal-free conditions or in the presence of zinc exhibited markedly reduced stability and substantially decreased purification yield (Fig. 1F). In agreement with these observations, quantitative analysis of iron and sulfur associated with SoxR revealed that SoxR isolated from cells grown in M9 medium without iron supplementation, as well as zinc-treated SoxR, contained significantly lower levels of Fe and S than SoxR isolated from cells grown under iron-supplemented conditions (Fig. 1G).

Collectively, these results indicate that zinc ions specifically target the [2Fe–2S] cluster of *K. pneumoniae* SoxR, leading to cofactor loss and a pronounced reduction in protein stability. Meanwhile, although SoxR isolated from cells grown in iron-depleted M9 medium remained detectable, its protein stability was markedly compromised, a defect that was further exacerbated in the presence of zinc ions.

### Zinc ions directly and dose dependently disrupt the [2Fe–2S] cluster of *K. pneumoniae* SoxR *in vitro* and accelerate protein precipitation

**Fig 2:**
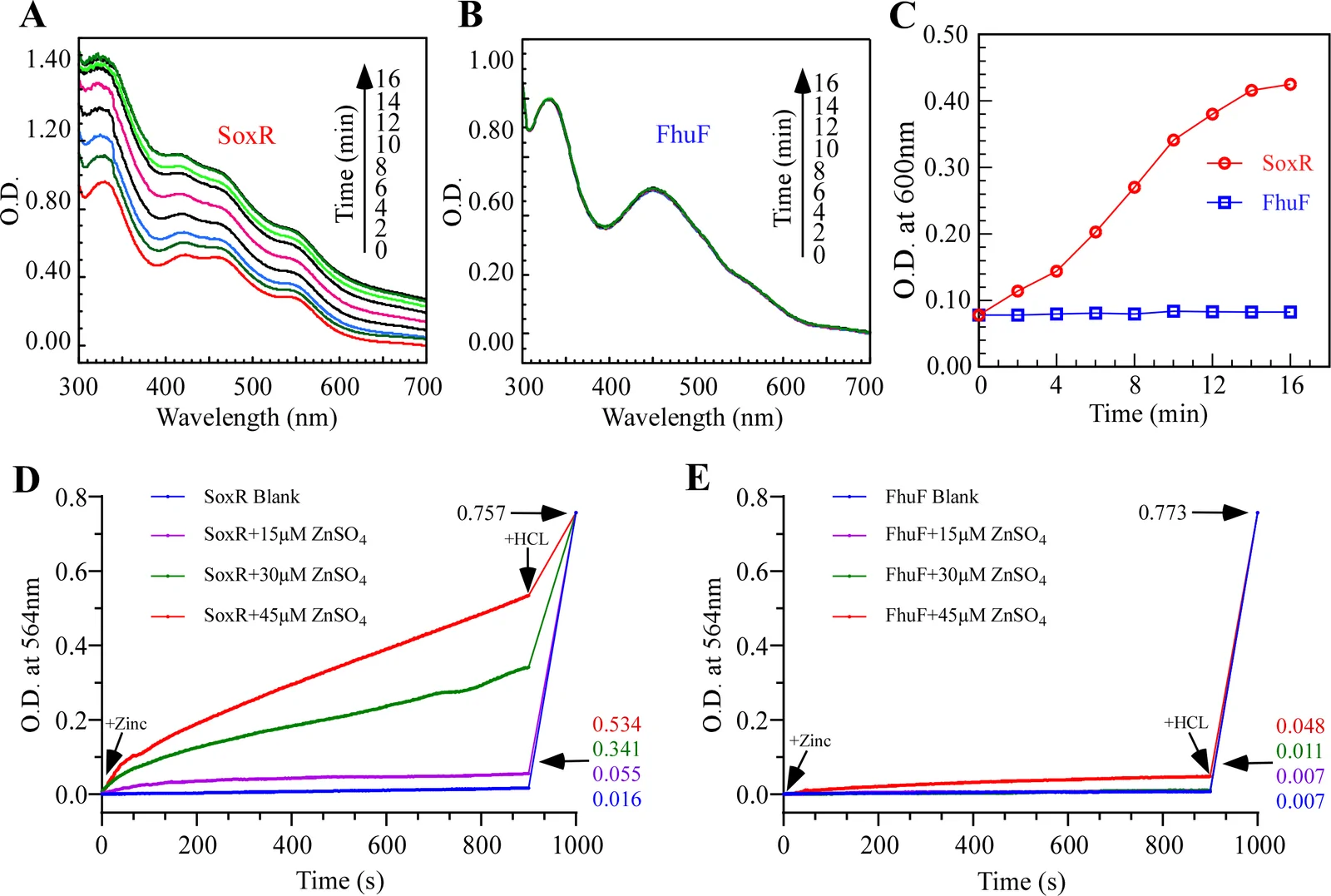
Zinc ions directly and dose-dependently disrupt the [2Fe–2S] cluster of *K. pneumoniae* SoxR *in vitro* and accelerate protein precipitation. (**A**) Time-resolved UV-visible absorption spectra of purified *K. pneumoniae* SoxR incubated *in vitro* with 15 μM ZnSO_4_ for 0–16 min. The final concentration of SoxR in the reaction system was fixed at 100 μM, and spectra were recorded over the wavelength range of 300–700 nm. (**B**) Time-resolved UV–visible absorption spectra of the purified control protein FhuF incubated with 15 μM ZnSO_4_ under identical conditions for 0–16 min. The final concentration of FhuF was likewise normalized to 100 μM. (**C**) Time-dependent changes in absorbance at 600 nm for SoxR (red) and FhuF (blue) in the presence of 15 μM ZnSO₄, reflecting alterations in solution turbidity. (**D**) Quantification of iron release from SoxR proteins treated with different concentrations of ZnSO_4_ (15, 30, and 45 μM) using the ferrozine-based colorimetric assay. Absorbance at 564 nm was monitored over time, and HCl was added at the end of the reaction to fully release residual iron as a control. (**E**) Ferrozine-based colorimetric analysis of iron release from the control protein FhuF treated with different concentrations of ZnSO₄ under the same experimental conditions. All experiments were performed at least three times, and the most representative results are shown.

Given that intracellular zinc ions were shown in Fig. 1 to markedly affect the Fe–S cluster status and protein stability of *K. pneumoniae* SoxR, we next evaluated the direct impact of zinc ions on preassembled SoxR [2Fe–2S] clusters in an *in vitro* system. To this end, purified SoxR proteins were incubated in Eppendorf tubes with 15 μM ZnSO₄, and time-resolved UV-visible absorption spectra were recorded. As shown in Fig. 2A, in the presence of ZnSO₄, the absorption spectra of SoxR changed markedly with increasing incubation time. Specifically, the overall optical density gradually increased, whereas the characteristic Fe–S cluster absorption peaks were progressively attenuated and eventually almost completely lost, indicating a concomitant increase in protein turbidity and disruption of the Fe–S cluster. In contrast, under identical experimental conditions, the UV-visible absorption spectra of the control protein *E. coli* FhuF, a [2Fe–2S] cluster protein, remained highly stable throughout the incubation period, with no obvious spectral alterations observed (Fig. 2B). Further quantitative analysis of absorbance at 600 nm revealed that, in the presence of 15 μM ZnSO₄, the OD_600_ of the SoxR solution increased continuously over time, whereas the OD_600_ of FhuF remained essentially constant (Fig. 2C). These results indicate that zinc ions specifically induce aggregation or precipitation of SoxR *in vitro*, an effect that is not universally observed among Fe–S proteins.

To further verify the dose dependence of zinc-mediated disruption of the SoxR Fe–S cluster, free iron released during ZnSO_4_ treatment was quantified using a ferrozine-based colorimetric assay. As shown in Fig. 2D, increasing the ZnSO₄ concentration from 15 to 30 μM and 45 μM led to a pronounced increase in absorbance at 564 nm in the SoxR system, indicating that higher zinc concentrations further promoted Fe–S cluster dissociation and iron release. Upon addition of HCl at the end of the reaction to fully release iron from the protein, all groups exhibited a sharp increase in absorbance, confirming the detectability of iron within the system. By contrast, in the control protein FhuF, absorbance at 564 nm remained at a very low level even under higher ZnSO_4_ concentrations, with no significant differences observed among the different zinc treatments (Fig. 2E).

Overall, the *in vivo* and *in vitro* results clearly demonstrate that zinc ions directly and specifically target *K. pneumoniae* SoxR and competitively disrupt its [2Fe–2S] cluster in a dose-dependent manner, thereby leading to Fe–S cofactor dissociation and a pronounced reduction in protein stability. This behavior is highly consistent with the zinc responsiveness of *E. coli* SoxR, suggesting that the specific response of SoxR to zinc ions is evolutionarily conserved across species.

### Zinc ions in combination with the superoxide inducer vitamin K3 inhibit the growth of *K. pneumoniae* through superoxide stress

**Fig 3:**
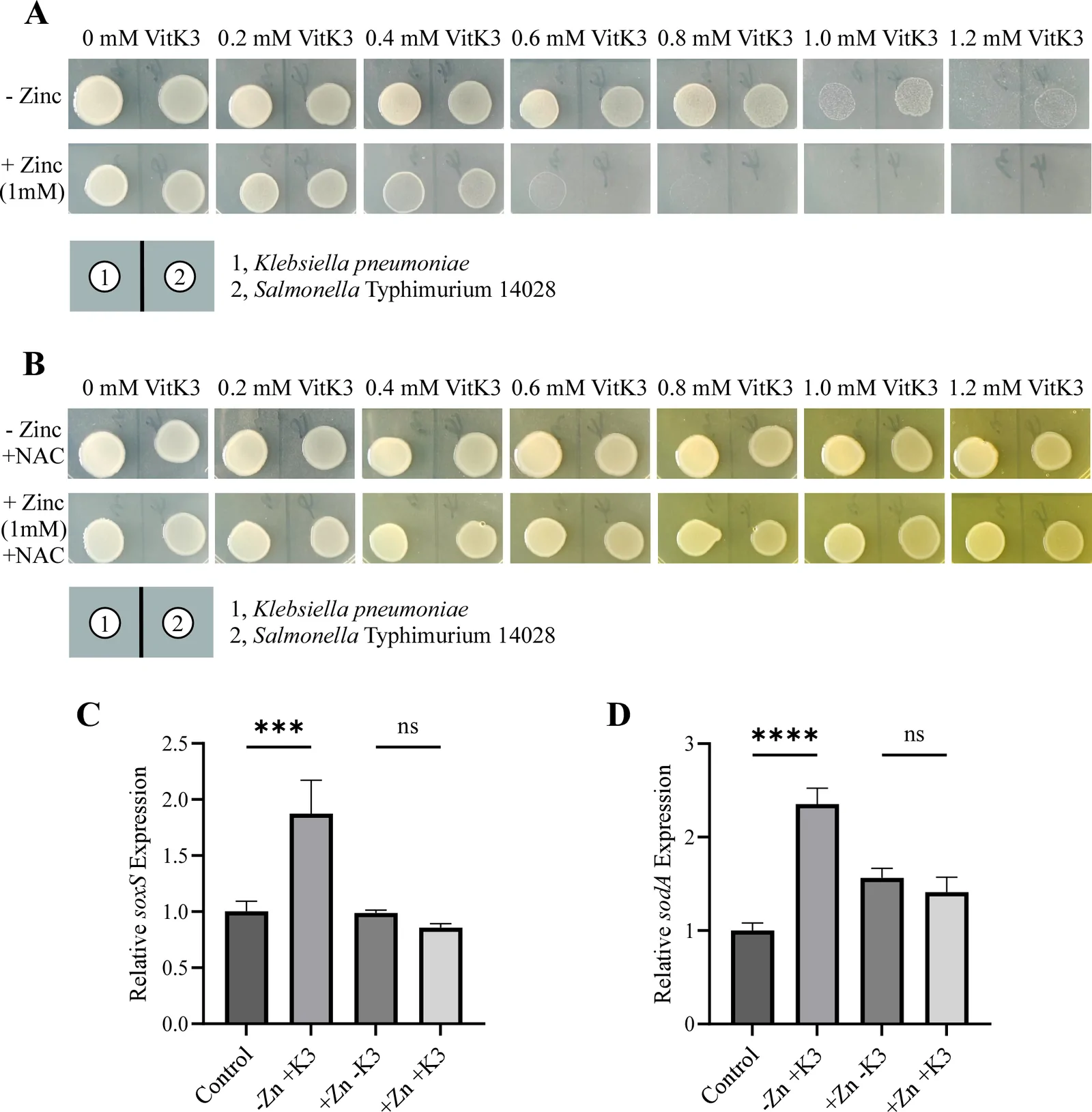
Zinc ions in combination with the superoxide inducer vitamin K3 inhibit the growth of *K. pneumoniae* through superoxide stress. (**A**) Growth of *K. pneumoniae* and *Salmonella enterica* serovar Typhimurium on LB agar plates supplemented with increasing concentrations of vitamin K3 (VitK3), in the absence or presence of zinc ions (1 mM). Bacterial spots were inoculated separately on the same plate, where 1 denotes *K. pneumoniae* and 2 denotes *S*. Typhimurium. (**B**) Growth of *K. pneumoniae* and *S*. Typhimurium on LB agar plates supplemented with 5 mM N-acetylcysteine (NAC), comparing different concentrations of VitK3 in the absence or presence of zinc ions (1 mM). (**C**) Quantitative analysis of the relative expression levels of the *soxS* gene in *K. pneumoniae* under different treatment conditions. (**D**) Quantitative analysis of the relative expression levels of the *sodA* gene in *K. pneumoniae* under different treatment conditions. All experiments were performed at least three times, and the most representative results are shown. *P < 0.05, **P < 0.01, ***P < 0.001, ****P < 0.0001; ns indicates no significant difference.

Given that *K. pneumoniae* and *S*. Typhimurium both belong to the Enterobacteriaceae family and represent clinically relevant enteric pathogens and given that their SoxR proteins share high similarity at both the sequence and functional levels (36), we evaluated the antibacterial applicability of zinc ions combined with a superoxide inducer using a side-by-side plate-based assay. We first compared the effects of increasing concentrations of vitamin K3 on the growth of the two bacterial species on lysogeny broth (LB) agar plates, either in the absence or presence of zinc ions. As shown in Fig. 3A, in the absence of zinc ions, vitamin K3 inhibited the growth of *K. pneumoniae* and *S*. Typhimurium only at relatively high concentrations. In contrast, upon zinc supplementation, growth of both bacteria was markedly restricted at substantially lower concentrations of vitamin K3, indicating that zinc ions significantly potentiated the inhibitory effect of vitamin K3 against Enterobacteriaceae. Furthermore, to verify the specificity of zinc in this synergistic strategy, we evaluated copper as a control metal. Unlike zinc, the combination of 1 mM copper with vitamin K3 failed to induce any synergistic growth inhibition against either *K. pneumoniae* or *S*. Typhimurium (Fig. S2), confirming that the observed synergistic lethality is uniquely driven by zinc.

To determine whether the observed growth inhibition depended on oxidative stress, the radical scavenger N-acetylcysteine (NAC) was introduced into the plate-based system. As shown in Fig. 3B, in the presence of NAC, vitamin K3 failed to exert a detectable inhibitory effect on the growth of *K. pneumoniae* or *S.* Typhimurium across the entire concentration range tested, regardless of zinc supplementation. Notably, bacterial growth remained largely unaffected even at elevated vitamin K3 concentrations, suggesting that the antibacterial effect elicited by the zinc–vitamin K3 combination was dependent on reactive oxygen species, particularly superoxide accumulation.

To validate these observations at the molecular level, we further examined the expression of representative antioxidative genes regulated by SoxR in *K. pneumoniae*. As shown in Fig. 3C and D, treatment with vitamin K3 alone significantly upregulated the expression of *soxS* and *sodA*, indicating that the exogenous superoxide inducer activated the SoxR-mediated oxidative stress response. However, under combined zinc and vitamin K3 treatment, the expression levels of *soxS* and *sodA* did not increase significantly, indicating that SoxR was no longer able to effectively sense or respond to superoxide signals following zinc exposure. Collectively, these results demonstrate that zinc ions impair the function of SoxR in *K. pneumoniae*, thereby suppressing SoxR-mediated antioxidative defense pathways. As a consequence, bacteria fail to induce high-level expression of *soxS* and *sodA* under vitamin K3-induced superoxide stress, ultimately resulting in redox imbalance and growth inhibition.

Together, these findings validate, at both the phenotypic and molecular levels, the feasibility of zinc ions combined with vitamin K3 as a novel antibacterial strategy against *K. pneumoniae* and other clinically relevant bacteria.

### Evaluation of the bactericidal kinetics, biofilm formation, and resistance development of *K. pneumoniae* in response to zinc ions and vitamin K3 combination treatment

**Fig 4:**
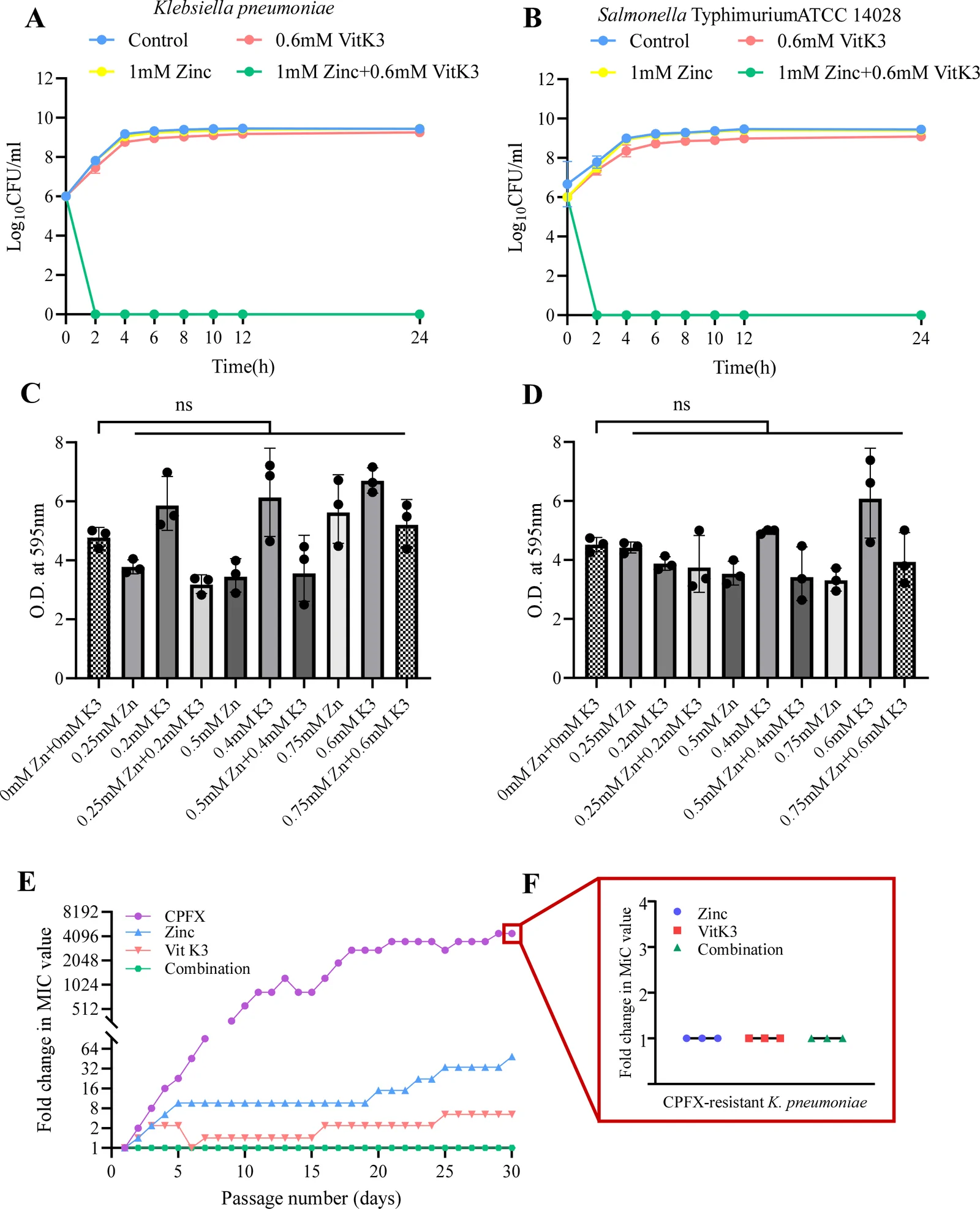
Evaluation of the bactericidal kinetics, biofilm formation, and resistance development of *K. pneumoniae* in response to zinc ions and vitamin K3 combination treatment. (**A and B**) Time–kill curves of *K. pneumoniae* (**A**) and *Salmonella* Typhimurium ATCC 14028 (**B**) treated with zinc ions (1 mM), vitamin K3 (0.6 mM), or their combination. (**C and D**) Effects of zinc ions (0.25, 0.5, and 0.75 mM), vitamin K3 (0.2, 0.4, and 0.6 mM), and their corresponding combinations on biofilm formation by *K. pneumoniae* (**C**) and *S.* Typhimurium (**D**) under sub-inhibitory conditions, as quantified by crystal violet staining at 595 nm. (**E**) Changes in minimum inhibitory concentration (MIC) values of *K. pneumoniae* during 30 consecutive passages under sub-MIC conditions of ciprofloxacin (CPFX), zinc ions, vitamin K3, or their combination. (**F**) Susceptibility of CPFX-resistant *K. pneumoniae* to zinc ions, vitamin K3, and their combination. All experiments were performed at least three times, and the most representative results are shown. *P < 0.05, **P < 0.01, ***P < 0.001, ****P < 0.0001*;* ns indicates no significant difference.

To evaluate the *in vitro* antibacterial activity of the zinc ion and vitamin K3 combination strategy, time–kill curve assays were performed using *K. pneumoniae* and *S.* Typhimurium (Fig. 4A and B). The results showed that treatment with either zinc ions at 1 mM or vitamin K3 at 0.6 mM alone did not exert a pronounced inhibitory effect on the growth of either bacterial species. In contrast, under combination treatment, bacterial counts rapidly declined within 2 h and reached complete eradication, exhibiting a pronounced and sustained bactericidal effect. These results indicated a strong synergistic antibacterial interaction between zinc ions and vitamin K3. Based on these observations, and to exclude the possibility that the antibacterial effect resulted from nonspecific suppression of virulence-associated phenotypes, biofilm formation was further evaluated under sub-inhibitory conditions (Fig. 4C and D). Zinc ions at 0.25, 0.50, and 0.75 mM, vitamin K3 at 0.20, 0.40, and 0.60 mM, as well as their corresponding combinations, were selected for treatment. The results demonstrated that biofilm formation levels did not differ significantly among any of the treatment groups, whether treated individually or in combination. These findings suggested that, at sub-inhibitory concentrations, the zinc–vitamin K3 combination strategy did not affect virulence-associated phenotypes represented by biofilm formation. Instead, its antibacterial activity was more likely attributable to disruption of bacterial redox homeostasis rather than suppression of virulence factors.

Given that prolonged exposure of bacteria to sublethal concentrations of antimicrobial agents can promote the emergence of resistance, a serial passaging assay under sub-MIC conditions was further performed to assess the potential resistance risk associated with the zinc–vitamin K3 combination strategy (Fig. 4E). The results showed that, after 30 consecutive passages under sublethal ciprofloxacin (CPFX) treatment, the MIC value of *K. pneumoniae* increased by approximately 4,096-fold relative to the initial level. In contrast, no notable increase in MIC values was observed throughout the passaging process in the zinc ion, vitamin K3, or combination treatment groups, indicating that this strategy was unlikely to induce bacterial resistance. Further analysis revealed that the zinc–vitamin K3 combination retained pronounced antibacterial activity against CPFX-resistant *K. pneumoniae* (Fig. 4F), suggesting the absence of cross-resistance between this strategy and fluoroquinolone antibiotics.

Collectively, the combined application of zinc ions and vitamin K3 exerted potent bactericidal activity without affecting virulence-associated phenotypes and exhibited a low risk of resistance development, highlighting its promising anti-resistance potential.

### *In vivo* protective effects of the zinc–vitamin K3 combination strategy in a *K. pneumoniae* infection model using *Galleria mellonella*

**Fig 5:**
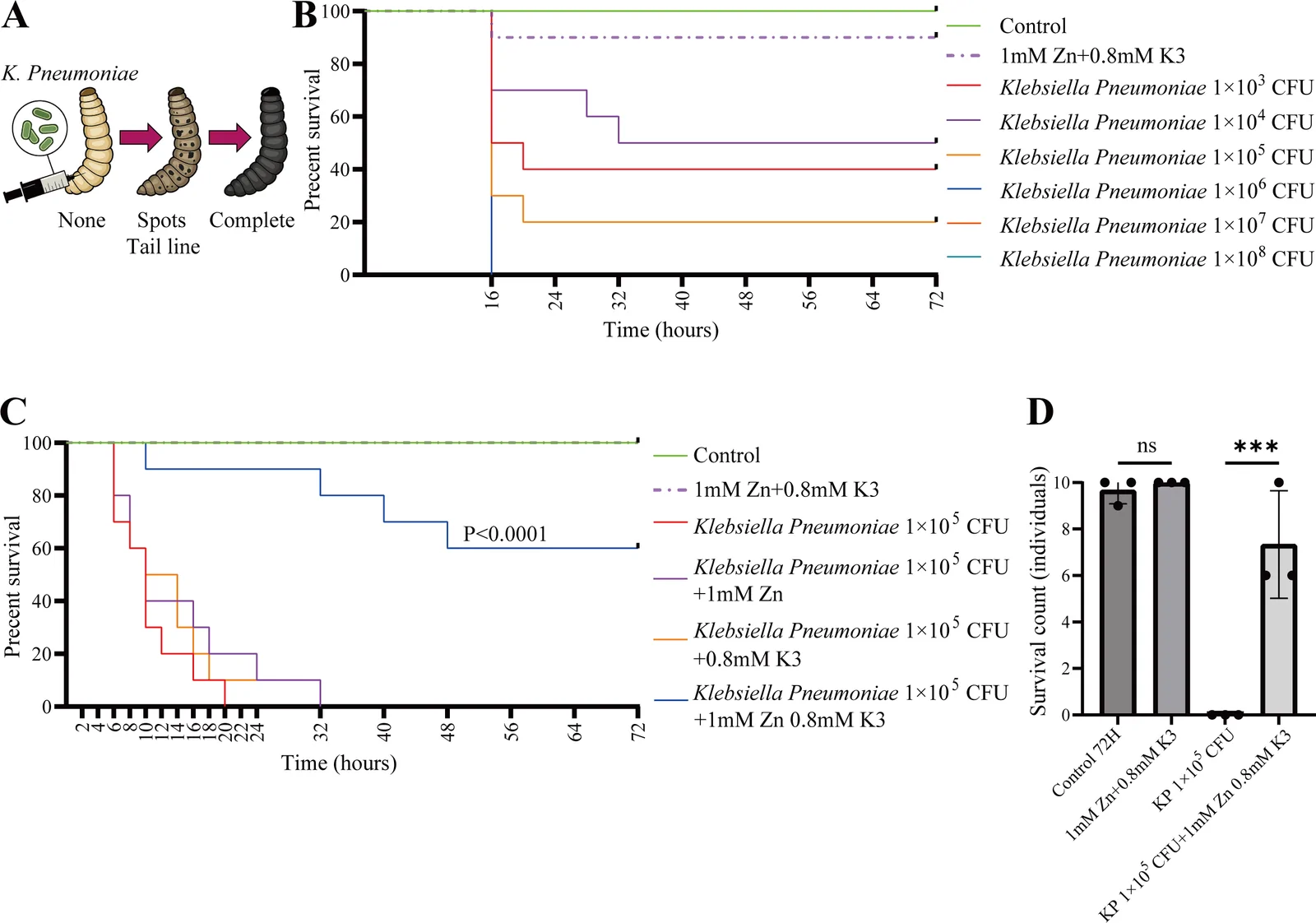
*In vivo* protective effects of the zinc–vitamin K3 combination strategy in a *K. pneumoniae* infection model using *Galleria mellonella*. (**A**) Schematic illustration of the acute *Galleria mellonella* infection model. *K. pneumoniae* was injected into the larval hemolymph via the posterior proleg, resulting in a progressive infection characterized by localized melanization, systemic melanization, and eventual death. (**B**) Survival curves of *G. mellonella* larvae infected with increasing doses of *K. pneumoniae* (1 × 10^3^ to 1 × 10^8^ CFU; *n* = 10 per group). (**C**) Effects of zinc ions (1 mM), vitamin K3 (0.8 mM), or their combination on the survival of *G. mellonella* larvae infected with 1 × 10^5^ CFU *K. pneumoniae* (*n* = 10 per group). (**D**) Statistical analysis of larval survival at 72 h across three independent experiments. All experiments were performed at least three times, and the most representative results are shown. *P < 0.05, **P < 0.01, ***P < 0.001, ****P < 0.0001*;* ns indicates no significant difference.

Given that the mechanistic analyses and resistance studies in this work were centered on *K. pneumoniae*, a pathogen of high clinical relevance, this organism was selected as the representative pathogen for subsequent *in vivo* validation using an acute *G. mellonella* infection model to evaluate the host-level efficacy of the zinc–vitamin K3 combination strategy. To further validate the antibacterial efficacy of the zinc–vitamin K3 combination strategy at the *in vivo* level, an acute *G. mellonella* infection model with *K. pneumoniae* was established (Fig. 5A). Following injection of *K. pneumoniae* into the larval hemolymph via the posterior proleg, larvae progressively transitioned from a healthy state to intermediate phenotypes, including localized melanized spots or posterior melanization, and subsequently developed systemic melanization culminating in death.

Initially, the pathogenicity of *K. pneumoniae* in the *G. mellonella* model was assessed (Fig. 5B). A microsyringe was used to inject 10 μL of bacterial suspensions containing different inoculum sizes (1 × 10^3^ to 1 × 10^8^ CFU) into the larval hemolymph (*n* = 10 per group), and larval survival was monitored over time. The results showed that larval survival decreased markedly with increasing bacterial inoculum. Based on the observed mortality kinetics, an inoculum of 1 × 10^5^ CFU was selected for subsequent treatment experiments. Accordingly, the effects of zinc ions (1 mM) and vitamin K3 (0.8 mM), either alone or in combination, on the survival of infected larvae were evaluated (Fig. 5C). The results showed that, under infection with 1 × 10⁵ CFU *K. pneumoniae*, larvae treated with zinc ions or vitamin K3 alone exhibited survival curves comparable to those of the infected control group, with nearly complete mortality within 24 h. In contrast, combined treatment with zinc ions and vitamin K3 significantly improved larval survival, demonstrating a pronounced protective effect (*P* < 0.0001). A statistical analysis of representative groups at the 72 h time point from three independent experiments was further performed (Fig. 5D). The results demonstrated that larval survival in the zinc–vitamin K3 combination group was significantly higher than that in the infected control and single-treatment groups, whereas no significant difference was observed between the uninfected control group and the combination-treated group.

Taken together, these findings indicated that the combination antibacterial strategy did not exert overt toxicity toward *G. mellonella* larvae and conferred robust anti-infective and host-protective effects in the *in vivo* infection model.

## DISCUSSION

**Fig 6:**
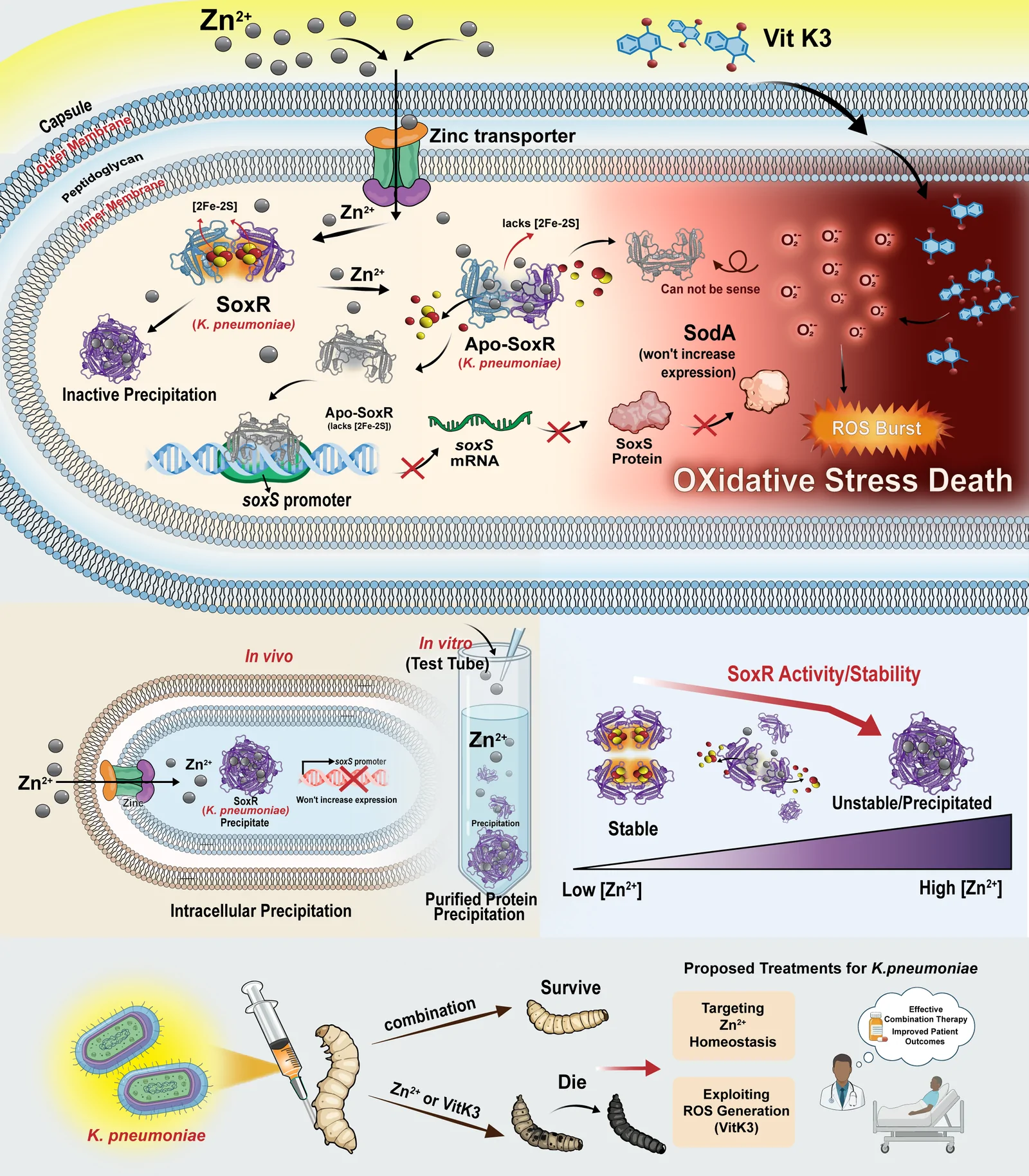
Proposed mechanistic model of zinc-mediated redox disruption and synergistic killing in *K. pneumoniae*. Zinc ions competitively target and disrupt the [2Fe–2S] cluster of the redox-sensing regulator SoxR, leading to structural destabilization and intracellular protein precipitation. This metal-induced inactivation fundamentally paralyzes the downstream soxS–sodA antioxidant defense axis. Consequently, the bacteria become exquisitely vulnerable to ROS bursts (superoxide stress) generated by vitamin K3. This Zn²^+^/vitamin K3 synergistic strategy leads to robust oxidative stress-induced death and confers significant *in vivo* protective efficacy against *K. pneumoniae* infection, highlighting the therapeutic potential of targeting Fe–S cluster homeostasis.

Against the backdrop of a persistent decline in antibacterial drug innovation and the rapid global spread of drug-resistant gram-negative pathogens, there is an urgent need to explore novel antibacterial strategies that target intrinsic physiological vulnerabilities of bacteria rather than conventional bactericidal or bacteriostatic targets (37–39). In this study, *K. pneumoniae*, a clinically important pathogen, was investigated to systematically elucidate the molecular mechanism by which zinc ions exert antibacterial activity through targeting the SoxR-mediated oxidative stress defense pathway. In doing so, the vulnerability associated with Fe–S cluster integrity and redox regulation was extended from the model organism *E. coli* to a major clinical pathogen (35). Our results demonstrate that SoxR in *K. pneumoniae* is likewise highly sensitive to zinc. Zinc overload markedly compromises SoxR structural stability, competitively disrupts the integrity of its [2Fe–2S] cluster, and suppresses the stress-induced upregulation of downstream *soxS* and *sodA* transcription, thereby weakening bacterial defense against superoxide stress. Furthermore, we demonstrate that this zinc-induced, molecular-level defect in oxidative defense can be effectively amplified by the classical superoxide-generating agent vitamin K3, thereby transforming SoxR inactivation from an isolated regulatory impairment into a tractable and amplifiable antibacterial effect (Fig. 6). Collectively, this study not only defines the functional significance of the zinc–SoxR–oxidative stress axis in *K. pneumoniae* but also provides new experimental evidence and a conceptual framework for nontraditional antibacterial strategies based on perturbation of endogenous bacterial redox homeostasis.

Although SoxR was initially and most extensively characterized in *E. coli*, as a redox-sensitive transcription factor harboring a [2Fe–2S] cluster, it is in fact widely distributed among diverse bacterial species and participates in the regulation of oxidative stress responses, metabolic remodeling, and antibiotic resistance-associated processes (40, 41). Within the Enterobacteriaceae, the SoxR/SoxS pathway is closely linked not only to superoxide defense but also to multidrug resistance development through regulation of efflux pump systems, as suggested by resistance phenotype studies in *Salmonella* and *K. pneumoniae* (25, 42). However, in contrast to *E. coli*, the metal sensitivity, structural stability, and specific role of SoxR in metal–oxidative stress crosstalk in *K. pneumoniae* have remained largely unexplored. The present study provides functional evidence that the SoxR–SoxS–SodA axis constitutes a bona fide and regulatable oxidative stress defense pathway in *K. pneumoniae*. This finding is highly consistent with transcriptomic and genetic studies identifying SoxS as a central regulator of oxidative stress responses and antibiotic resistance in *K. pneumoniae* (18) and further enriches the molecular basis of SoxRS-mediated control of bacterial survival and adaptation. Moreover, previous studies have shown that the SoxRS pathway can act in concert with other stress regulatory networks, such as RpoS and c-di-GMP signaling, to influence virulence, biofilm formation, and *in vivo* fitness of *K. pneumoniae* (43), suggesting that SoxR dysfunction may result in multilayered physiological consequences. On this basis, our study further demonstrates the functional evolutionary conservation of SoxR as an Fe–S cluster protein among Enterobacteriaceae and, for the first time, explicitly incorporates the regulatory paradigm of metal ion–mediated disruption of SoxR structural stability into the physiology and pathogenesis of *K. pneumoniae*.

From a molecular mechanistic perspective, the inhibitory effect of zinc on SoxR function observed in this study is more likely attributable to metal competition and mismetallation, rather than to indirect disruption of Fe–S clusters through zinc-induced oxidative stress. Zinc ions themselves lack intrinsic redox activity, and their antibacterial effects are therefore typically associated with perturbations in metal homeostasis, protein structural destabilization, and improper metal coordination, rather than with classical mechanisms involving direct oxidation by reactive oxygen species (44, 45). The pronounced sensitivity of SoxR to zinc is likely linked to the structural features of its [2Fe–2S] cluster. This cluster is coordinated by multiple cysteine residues and adopts a relatively exposed conformation, rendering it highly susceptible to interference by exogenous metal ions. Classical mutagenesis studies have demonstrated that substitution of any critical cysteine residue in SoxR results in loss of the [2Fe–2S] cluster and concomitant protein inactivation (46). Consistent with our *in vitro* and *in vivo* evidence obtained in *K. pneumoniae*, zinc ions are more likely to competitively bind these cysteine coordination sites, thereby interfering with proper Fe–S cluster assembly or stability and ultimately impairing SoxR-mediated redox sensing and transcriptional activation. Accordingly, SoxR is not merely a passive sensor of oxidative signals but rather represents a highly vulnerable molecular node at the intersection of metal homeostasis and redox regulation. Targeted disruption of the SoxR [2Fe–2S] cluster by zinc therefore provides a mechanistic foundation for translating this molecular vulnerability into a tractable antibacterial strategy.

The combination of Zn²^+^ and vitamin K3 does not represent a simple additive antibacterial effect; instead, these agents act on two distinct but interdependent dimensions within the same redox defense pathway, namely, the defense threshold and the imposed stress intensity, thereby generating a synergistic amplification effect. This combination exemplifies a mechanism-driven antibacterial design rather than empirical drug pairing. In *K. pneumoniae*, zinc is an essential yet tightly regulated trace metal, with its homeostasis maintained through finely tuned high-affinity uptake and efflux systems; disruption of this balance markedly compromises bacterial tolerance to multiple environmental stresses, including oxidative stress (29, 47). However, zinc applied alone is often partially buffered by bacterial metal homeostasis networks, which consequently limits its antibacterial efficacy. The present study demonstrates that Zn²^+^ in *K. pneumoniae* specifically destabilizes the SoxR [2Fe–2S] cluster, weakens the SoxR–SoxS–SodA–mediated superoxide defense axis, and thereby substantially lowers the bacterial defense threshold against oxidative pressure. In this context, introduction of the classical superoxide-generating agent vitamin K3 markedly elevates intracellular superoxide burden, imposing sustained pressure on the oxidative defense system. Previous studies have shown that vitamin K3 is capable of inducing reactive oxygen species generation, disrupting essential bacterial physiological processes, and potentiating antibacterial activity (48, 49). Consequently, once the regulatory node of oxidative stress defense has been weakened by Zn²^+^, the superoxide pressure imposed by vitamin K3 can no longer be effectively buffered but is instead amplified into lethal oxidative damage. Acting in concert at SoxR, a convergence point of redox and metal regulation, these two agents establish a mechanism-driven and potentially scalable nontraditional antibacterial strategy. Moreover, this nontraditional antibacterial strategy appears less prone to resistance development, underscoring its potential value for future clinical application. However, considering that vitamin K3 is a synthetic derivative with known toxicity in humans, future investigations should evaluate the synergistic efficacy of clinically viable analogs, such as vitamin K1 (phylloquinone) or K2 (menaquinone), to further enhance the translational impact of this strategy (50).

Here, we introduce the acute *Galleria mellonella* infection model to evaluate the *in vivo* antibacterial efficacy and host safety of the Zn²^+^/vitamin K3 combination, serving as an important transitional platform linking *in vitro* mechanistic studies with higher-order animal models. *G. mellonella* offers several practical advantages, including a well-defined anatomy, experimental simplicity, low cost, and minimal ethical constraints; moreover, its innate immune system exhibits functional similarities to that of vertebrates, which has led to its widespread use in evaluating bacterial virulence, *in vivo* antimicrobial efficacy, and potential host toxicity (51, 52). Accumulating evidence indicates that virulence phenotypes of different bacterial strains or mutants in *G. mellonella* frequently correlate well with outcomes observed in mammalian models, such as mice, making this system particularly suitable for early-stage strategy screening and *in vivo* feasibility assessment (53–55). In the present study, combined treatment with Zn²^+^ and vitamin K3 significantly improved host survival in the *G. mellonella* infection model, whereas monotherapy did not confer comparable protection. Notably, no apparent host toxicity was observed within the tested dosage range, providing *in vivo* support for our proposed mechanistic model.

Although this study systematically elucidates the molecular mechanism by which Zn²^+^ disrupts SoxR [2Fe–2S] cluster stability and compromises oxidative stress defense in *K. pneumoniae*, several aspects warrant further investigation. First, high-resolution structural information describing the interaction between Zn²^+^ and SoxR is currently lacking, and the precise conformational basis by which Zn²^+^ competes for [2Fe–2S] cluster coordination remains to be elucidated using structural biology approaches. Second, although the *G. mellonella* model provides valuable *in vivo* validation, the efficacy and safety of the Zn²^+^/vitamin K3 combination in mammalian infection models require further evaluation to better approximate clinical scenarios. In addition, beyond *E. coli* and *K. pneumoniae* examined in our parallel studies, the applicability of this strategy to other clinically relevant gram-negative pathogens remains to be systematically explored. Third, recognizing the extreme genomic and phenotypic diversity of *K. pneumoniae*, the current mechanistic validation was primarily conducted using a representative clinical isolate. To facilitate clinical translation, it is imperative for future studies to systematically screen the susceptibility to the Zn²^+^/vitamin K3 combination across a broad spectrum of clinical isolates, particularly encompassing diverse multilocus sequence typing (MLST) lineages, hypervirulent *K. pneumoniae,* and various multidrug-resistant or extensively drug-resistant strains. Furthermore, intervention strategies targeting SoxR and other Fe–S cluster–regulated proteins may complement conventional antibiotics or serve as antimicrobial potentiators when used in combination therapies (56–58). Systematic evaluation of synergistic or antagonistic interactions between this combination and existing antibiotics, together with assessment of long-term safety in complex host environments, will be critical for advancing this nontraditional antibacterial strategy toward clinical translation (59, 60).

Mechanistic analyses in this study focus primarily on *K. pneumoniae*, while *Salmonella enterica* serovar Typhimurium was included in selected *in vitro* experiments to preliminarily assess the broader antibacterial potential of the Zn²^+^/vitamin K3 combination within clinically relevant Enterobacteriaceae. Given the high degree of conservation in oxidative stress responses and redox regulatory systems between these organisms, this design supports the potential broad applicability of the combined strategy without deviating from the central mechanistic framework of the study.

In summary, this study demonstrates that the SoxR-dependent Fe–S cluster-based redox regulatory network in *K. pneumoniae* constitutes a critical vulnerability in bacterial adaptation to metal and oxidative stress. By weakening oxidative defense through Zn²^+^ and amplifying superoxide stress with vitamin K3, this molecular defect can be converted into a highly effective antibacterial outcome with a reduced propensity for resistance development. Notably, this strategy does not rely on classical targets that directly inhibit bacterial growth; instead, it suppresses bacterial survival by disrupting Fe–S clusters and inducing stress homeostasis imbalance, thereby offering a potential means to reduce selective pressure for resistance. Collectively, this work extends metal–redox homeostasis-based intervention from *E. coli* to a major clinical pathogen and provides both experimental evidence and a translational framework for the development of nontraditional antibacterial strategies targeting bacterial stress defense systems.

## MATERIALS AND METHODS

### Strains and plasmid construction

The *Klebsiella pneumoniae* strain used in this study was originally isolated from a clinical sample in a tertiary teaching hospital (Wenzhou, China) during routine diagnostic procedures. The strain has been archived in our laboratory collection, and its identity was confirmed by 16S rRNA gene sequencing. *Salmonella enterica* serovar Typhimurium (ATCC 14028) was purchased from the American Type Culture Collection (ATCC). *Escherichia coli* MC4100, obtained from the China General Microbiological Culture Collection Center, served as the host for plasmid maintenance and recombinant protein expression. The empty pBAD vector and the cold-shock expression vector pCold I were purchased from Takara Bio Inc. (Kusatsu, Japan). For the construction of arabinose-inducible expression plasmids, the coding sequences of *soxS* and *fhuF* were amplified from the *K. pneumoniae* genome via PCR. The resulting DNA fragments were inserted into the pBAD vector using a one-step cloning kit (C112-01, Vazyme Biotech, Nanjing, China) to generate pBAD-SoxS and pBAD-FhuF. All constructed plasmids were verified by DNA sequencing and subsequently transformed into *E. coli* MC4100 cells for further experiments.

### Gene knockout

The soxR genes were deleted from the *E. coli* MC4100 wild-type strain using a previously established protocol developed in our laboratory (61).

### Bioinformatic analysis

To evaluate the evolutionary conservation of SoxR, the amino acid sequences of SoxR proteins from *E. coli*, *K. pneumoniae*, and *S.* Typhimurium were subjected to comparative analysis. Multiple sequence alignment was performed using SnapGene software (version 6.0.2; GSL Biotech LLC) with the integrated Multiple Sequence Comparison by Log-Expectation (MUSCLE) algorithm. Particular emphasis was placed on identifying the four putative cysteine residues essential for the coordination of the [2Fe–2S] cluster. The conservation of these residues was assessed to determine the structural and functional consistency of the redox-sensing domain across the three species.

### Protein expression and purification

Protein expression and purification were conducted according to the protocols previously established in our laboratory with specific modifications (33, 62, 63). Briefly, *E. coli* MC4100 cells harboring the expression plasmids were grown in LB or M9 minimal medium at 37°C and 250 rpm until the OD_600_ reached 0.6. In LB medium, three groups were established to assess zinc toxicity: a control group, a pre-induction group (1 mM ZnSO_4_ added 10 min before induction), and a post-induction group. For the post-induction group, cells were washed to remove the inducer and resuspended in fresh LB containing 1 mM ZnSO_4_ and 34 μg/μL chloramphenicol to halt new protein synthesis. In M9 medium (64), cultures were supplemented with either 25 μM ferric citrate or 25 μM ZnSO_4_. Protein expression was induced with L-arabinose at a 1:5,000 dilution for LB and 1:2,000 for M9 for 3 h.

All purification steps were performed under anaerobic conditions. Buffers (20 mM Tris-HCl, 500 mM NaCl, pH 8.0) were supplemented with 2 mM DTT and purged with pure argon gas for 10 min to remove dissolved oxygen. Cells were harvested by centrifugation (4,000 rpm, 10 min, 4°C) and lysed using a high-pressure cell homogenizer. The lysate was cleared by centrifugation at 22,000 rpm for 40 min at 4°C. The resulting supernatant was filtered (0.22 μm) and purified using Ni-NTA affinity chromatography on an AKTA system. Purified proteins were desalted, and their UV-visible absorption spectra (250–700 nm) were recorded using a U3900 spectrophotometer.

### Protein solubility, purity, and Concentration

The solubility and purity of the recombinant protein were assessed by SDS-PAGE followed by Coomassie Brilliant Blue staining, using whole-cell lysates, culture supernatants, and purified protein fractions. A pre-stained protein ladder (Cat# WJ102, Yeasen Biotechnology [Shanghai] Co., Ltd., Shanghai, China) was used as a molecular weight marker. The concentration of the purified protein was determined by measuring the absorbance at 280 nm and calculating using the published extinction coefficient.

### Metal and sulfur content analyses

The iron content of purified protein samples was determined using the ferrozine-based colorimetric assay. In the presence of L-cysteine, iron ions released from the protein reacted with ferrozine to form a magenta complex with a characteristic absorbance peak at 564 nm. The total sulfur content was quantified by reacting inorganic sulfide with FeCl_3_ and N,N-dimethyl-p-phenylenediamine to generate a blue compound, which was measured by its specific absorbance at 669 nm. All colorimetric measurements were performed using a U3900 spectrophotometer.

### *In vitro* zinc competition and iron release assays

To investigate the competitive disruption of SoxR [2Fe-2S] clusters by zinc ions, an *in vitro* iron release assay was performed using ferrozine as a chelating indicator. Purified SoxR proteins (100 μM) were incubated with varying concentrations of ZnSO_4_ (15, 30, or 45 μM) in a reaction system containing ferrozine. The displacement of iron by zinc was monitored in real-time by recording the increase in absorbance at 564 nm using a U3900 spectrophotometer. The control protein FhuF was treated under identical conditions to verify the specificity of the zinc-SoxR interaction. At the end of the reaction, concentrated HCl was added to the mixture to fully release all residual iron from the protein as a reference for total iron content.

### Reverse transcriptase quantitative PCR

Total RNA was extracted using the FreeZol Reagent RNA Isolation Kit (R711-01, Vazyme Biotech, Nanjing, China) and reverse-transcribed into cDNA using the Reverse Transcription RT Kit (11141ES60, Yeasen Biotechnology, Shanghai, China). Gene-specific primers were used for real-time PCR amplification. PCRs were performed in triplicate, with *rpoD* as the internal reference gene. The relative cDNA levels were determined using the comparative Ct method. Quantitative PCR primers were synthesized by GenScript Biotech, and their sequences are listed in Table 1.

**TABLE 1:** Primer list for reverse transcriptase quantitative PCR (qRT-PCR)

Primer name	Sequence 5′→3′	Product size (bp)
*Escherichia coli* MC4100		
*mdoG* (housekeeping gene)	F 5′-ATAACTATCGTCCGGAGTTGC-3′	124
*mdoG* (housekeeping gene)	R 5′-TTTTCCATGGAGAAGCTGCTG-3′	
*sodA*/F	F 5′-GGCGGTGGTTTCTACTGCTA-3′	80
*sodA*/R	R 5′-CCATAATCGGGAAGCCGGAA-3′	
*soxS*/F	F 5′-TGACGCATCAGACGCTTGGC-3′	87
*soxS*/R	R 5′-AAATCGGACGCTCGGTGGT-3′	
*Klebsiella pneumoniae*		
*rpoD* (housekeeping gene)	F 5′-TCTCCGGTACCGTTATCGAC-3′	201
*rpoD* (housekeeping gene)	R 5′-TGTCGAGCTTCTCAGCTTCA-3′	
*sodA*/F	F 5′-CCGCTGAAGAGCTGATTACC-3′	184
*sodA*/R	R 5′-TTGAAGTTCTCCACGGAACC-3′	
*soxS*/F	F 5′-GATGTTCCGTACCGTCATG-3′	145
*soxS*/R	R 5′-GAAGGTTTGCTGCGAGAC-3′	

### Plate growth assay

Individual colonies of *K. pneumoniae* and *S.* Typhimurium were isolated from LB agar and cultured overnight in 3 mL of LB broth. The resulting bacterial cells were washed three times with sterile saline and normalized to a final OD_600_ of 0.02. Subsequently, 2 μL aliquots of these suspensions were spotted onto LB agar plates supplemented with indicated concentrations of ZnSO_4_ and vitamin K3. The plates were incubated, and bacterial growth patterns were documented after 20 h.

### Time-kill curve assay

The bactericidal kinetics of the zinc and VitK3 combination were evaluated against *K. pneumoniae* and *S.* Typhimurium. Overnight cultures were adjusted to approximately 10^6^ CFU/mL in LB broth. Standardized suspensions were treated with 1 mM Zn^2+^, 0.6 mM VitK3, or their combination, while an untreated culture served as the control. At defined time points (0, 2, 4, 6, 8, 10, 12, and 24 h), viable counts were determined via serial dilution and spot-plating. Results were plotted as log10 CFU/mL versus time.

### Resistance development and cross-resistance assay

The potential for resistance development in *K. pneumoniae* was assessed via a 30-day serial passaging assay under sub-MIC concentrations of ciprofloxacin (CPFX), Zn^2+^, vitamin K3 (VitK3), or the Zn^2+^/VitK3 combination. MIC values for each group were determined daily throughout the passaging process. To evaluate cross-resistance, the susceptibility of the resulting CPFX-resistant mutants to Zn^2+^, VitK3, and the combination was determined using the broth microdilution method in LB medium (65).

### *Galleria mellonella* infection model

Larvae of *G. mellonella* were obtained from Tianjin Huiyude Biotechnology Co., Ltd. To assess virulence, larvae (*n* = 10 per group) were administered 10 μL of *K. pneumoniae* suspensions at dosages ranging from 1 × 10^3^ to 1 × 10^8^ CFU via the posterior proleg into the hemocoel. Following the analysis of mortality kinetics, a dose of 1 × 10^5^ CFU was selected for subsequent therapeutic evaluations. For treatment assays, larvae infected with 1 × 10^5^ CFU received a second 10 μL injection containing 1 mM Zn²^+^, 0.8 mM vitamin K3, the Zn²^+^/VitK3 combination, or a saline vehicle control. A separate group of uninfected larvae injected with saline served as the health control. Survival was tracked over a 72-h period; individuals were classified as dead if they exhibited systemic melanization and failed to respond to physical stimulation (66).

### Statistical analysis

All data are presented as mean ± standard deviation. Graphs were generated using GraphPad Prism 10.1.2, and figures were assembled in Adobe Illustrator 2024. Differences among multiple groups were assessed by one-way analysis of variance followed by Tukey’s *post hoc* test for multiple comparisons. A *P*-value of less than 0.05 was considered statistically significant.

## Data Availability

The data underlying this article are available in Zenodo at https://doi.org/10.5281/zenodo.19562771.

## SUPPLEMENTAL MATERIAL

Supplenental Material mbio.01146-26-s0001.docx Fig. S1 and S2.

ASM does not own the copyrights to Supplemental Material that may be linked to, or accessed through, an article. The authors have granted ASM a non-exclusive, world-wide license to publish the Supplemental Material files. Please contact the corresponding author directly for reuse.
