# Supplementary material for: Conserved vulnerability of SoxR underlies zinc-mediated redox disruption and synergistic killing in Klebsiella pneumoniae
Source: mBio. 2026 Jul 29;17(9):e01146-26. doi: 10.1128/mbio.01146-26 (PMC13556215; doi:10.1128/mbio.01146-26)
Supplement: Supplementary file 1 [file mbio.01146-26-s0001.docx]

**Supplementary Fig. S1. Quantitative analysis of SoxR-dependent transcriptional responses to paraquat (PQ). (A).** Quantitative analysis of the relative expression levels of the *soxS* (left) and *sodA* (right) genes in wild-type *Escherichia coli* MC4100 with or without PQ (100 μM) treatment. **(B).** Quantitative analysis of the relative expression levels of the *soxS* (left) and *sodA* (right) genes in the *soxR^-^* strain with or without PQ treatment. **(C).** Quantitative analysis of the relative expression levels of the *soxS* (left) and *sodA* (right) genes in the *soxR^-^* strain complemented with *E. coli soxR* or *Klebsiella pneumoniae* *soxR*, with or without PQ treatment. **(D).** Quantitative analysis of the relative expression levels of the *soxS* (left) and *sodA* (right) genes in *soxR^-^* strain complemented with *K. pneumoniae soxR* with or without PQ treatment. **P < 0.05*, ***P < 0.01*, ****P < 0.001*,*****P < 0.0001*; *ns* indicates no significant difference.

**Supplementary Fig. S2.** **Specificity of zinc in driving the synergistic bactericidal effect with vitamin K3.** Growth of *K. pneumoniae* and *Salmonella enterica* serovar Typhimurium on LB agar plates supplemented with increasing concentrations of vitamin K3 (VitK3), in the absence of metal ions, or the presence of zinc ions (1 mM) or copper ions (1 mM). Bacterial spots were inoculated separately on the same plate, where 1 denotes *K. pneumoniae* and 2 denotes *S.* Typhimurium.
